# Acriflavine, a Potent Inhibitor of HIF-1α, Disturbs Glucose Metabolism and Suppresses ATF4-Protective Pathways in Melanoma under Non-hypoxic Conditions

**DOI:** 10.3390/cancers13010102

**Published:** 2020-12-31

**Authors:** Román Martí-Díaz, María F. Montenegro, Juan Cabezas-Herrera, Colin R. Goding, José Neptuno Rodríguez-López, Luis Sánchez-del-Campo

**Affiliations:** 1 Department of Biochemistry and Molecular Biology A, School of Biology, IMIB-University of Murcia, 30100 Murcia, Spain; roman.marti@um.es (R.M.-D.); fermontenegro@um.es (M.F.M.); 2 Translational Cancer Research Group, University Hospital Virgen de la Arrixaca, IMIB, 30120 Murcia, Spain; juan.cabezas@carm.es; 3 Ludwig Institute for Cancer Research, Nuffield Department of Clinical Medicine, University of Oxford, Headington, Oxford OX3 7DQ, UK; colin.goding@ludwig.ox.ac.uk

**Keywords:** melanoma, acriflavine, MITF, HIF-1α, ATF4, glucose metabolism, oxygen homeostasis

## Abstract

**Simple Summary:**

Hypoxia is a common feature in solid tumors such as melanoma, contributing locally and systemically to tumor progression. Although the hypoxia response in tumor cells is well understood, the role of constitutively activated hypoxia-inducible factor (HIF)-1α in normoxic conditions is less known. Here, we used acriflavine, a chemical inhibitor of HIF-1α, to investigate the role of this transcription factor on the progression of melanoma under normoxic conditions. The data indicated that acriflavine disturbs glucose metabolism and induces melanoma cell death under normoxia. As a result, we describe a possible clinical option that may target melanoma cells irrespective of the hypoxic microenvironment of the tumors. However, the translational importance of these findings should be confirmed in pre-clinical models.

**Abstract:**

Hypoxia-inducible factor (HIF)-1α is constitutively expressed in melanoma cells under normoxic conditions and its elevated expression correlates with the aggressiveness of melanoma tumors. Here, we used acriflavine, a potent inhibitor of HIF-1α dimerization, as a tool to investigate whether HIF-1α-regulated pathways contribute to the growth of melanoma cells under normoxia. We observed that acriflavine differentially modulated HIF-1α-regulated targets in melanoma under normoxic conditions, although acriflavine treatment resulted in over-expression of vascular endothelial growth factor (VEGF), its action clearly downregulated the expression of pyruvate dehydrogenase kinase 1 (PDK1), a well-known target of HIF-1α. Consequently, downregulation of PDK1 by acrifavine resulted in reduced glucose availability and suppression of the Warburg effect in melanoma cells. In addition, by inhibiting the AKT and RSK2 phosphorylation, acriflavine also avoided protective pathways necessary for survival under conditions of oxidative stress. Interestingly, we show that acriflavine targets activating transcription factor 4 (ATF4) for proteasomal degradation while suppressing the expression of microphthalmia-associated transcription factor (MITF), a master regulator of melanocyte development and a melanoma oncogene. Since acriflavine treatment results in the consistent death of melanoma cells, our results suggest that inhibition of HIF-1α function in melanoma could open new avenues for the treatment of this deadly disease regardless of the hypoxic condition of the tumor.

## 1. Introduction

HIF-1 is a transcription factor that regulates the expression of genes linked to angiogenesis, cell differentiation, and anaerobic metabolism induced by hypoxia [1]. This transcription factor is believed to activate between 60 and 100 target genes. In addition to vascular endothelial growth factor (VEGF), HIF-1 controls the expression of several glycolytic enzymes and glucose transporters (such as GLUT1), required for elevated glucose uptake and metabolism [2]. HIF-1 thereby mediates the metabolic adaptation associated with conditions of reduced oxygen availability. As HIF-1 and the related factor HIF-2 regulate genes that play an important role in the progression of a wide range of tumors, modulating their activity could serve as a new approach in cancer therapeutics [3,4].

HIF-1 is a two subunit heterodimeric transcription factor made up of an α subunit (HIF-1α) and a β subunit (HIF-1β), both belonging to the family of transcription factors with bHLH/PAS domains (basic helix-loop-helix domain homologous to Per-ARNT-Sim) located at the N-terminus required for binding to DNA. HIF-1β is constitutively expressed in the nucleus; therefore, the activity of the HIF-1 complex is mainly mediated by the stability of the HIF-1α subunit. As with all proteins that play an important cellular function, the expression and activity of HIF-1 are subject to complex regulatory mechanisms that are directed, in this specific case, mainly to the HIF-1α subunit. Specifically, hydroxylation, ubiquitination, and acetylation control oxygen-dependent proteasome-mediated degradation of HIF-1α [5] with hydroxylation being especially important. Thus, under normoxic conditions, proline residues 402 and/or 564 are modified by prolyl hydroxylases (PHDs), allowing the recognition of HIF-1α by the Von Hippel–Lindau tumor suppressor that is part of an E3 ubiquitin protein ligase complex [5]. Conversely, under hypoxic conditions, HIF-1α is stabilized and translocates from the cytoplasm to the nucleus, where together with the HIF-1β protein forms the transcriptionally active form of HIF-1 that binds hypoxia response elements (HREs) in target genes. While the expression and function of HIF-1 under hypoxic conditions are well recognized, less is known about its function under normoxic conditions [6].

Hypoxic regions in solid tumors frequently give rise to a high growth tumor rate and allow for the generation of aberrant blood vessels [7]. Consequently, the risk of invasion, metastasis is increased by intratumor hypoxia, leading to elevated patient mortality [8]. However, while low oxygen clearly defines hypoxic domains within tumors, some tumors can activate the hypoxia response pathways even under normoxic conditions, a feature attributed to idiosyncratic metabolism. Melanoma, the most dangerous form of skin cancer, may represent one such tumor type [6]. Melanin-synthesis, a specialized differentiation-associated function of melanocytes, generates superoxide and hydrogen peroxide, which contribute to oxidative stress [9,10]. The mechanisms that allow melanocytes and melanoma cells to survive under this oxidative stress are unclear, however, some evidence points to the involvement of HIF-1α. Melanocytes respond to UV light by activating pro-survival pathways such as PI3K/AKT, CREB, and NFB, which ultimately lead to the expression of pro-survival proteins such as Bcl2 and the microphthalmia-associated transcription factor (MITF). Since MITF can activate HIF-1α transcription [11], it is plausible that a HIF-1-activated gene expression program would contribute to the resistance of melanocytes to oxidative stress [12]. Interestingly, it has been elegantly demonstrated that, in contrast to other tumor types, HIF activity can be constitutively activated in malignant melanoma cells even under normoxic conditions [13], and that elevated HIF-1α expression contributes to a harmful phenotype of human tumor cells under normoxic conditions [6]. Therefore, targeting HIF-1α function, regardless of the hypoxic condition of the tumor, may be a useful strategy to treat human melanoma.

Lee et al. [14] identified the antimicrobial drug acriflavine (ACF) as one of the most potent inhibitors of HIF-1 dimerization. Since the activity of ACF on melanoma cells has not been assayed in detail to date, here we examined the effect of this drug on the metabolism and progression of melanoma under normoxic expression of HIF-1α.

## 2. Results

### 2.1. Acriflavine Restricts Glucose Availability and Decreases Glycolysis in Melanoma Cells

**Figure 1:**
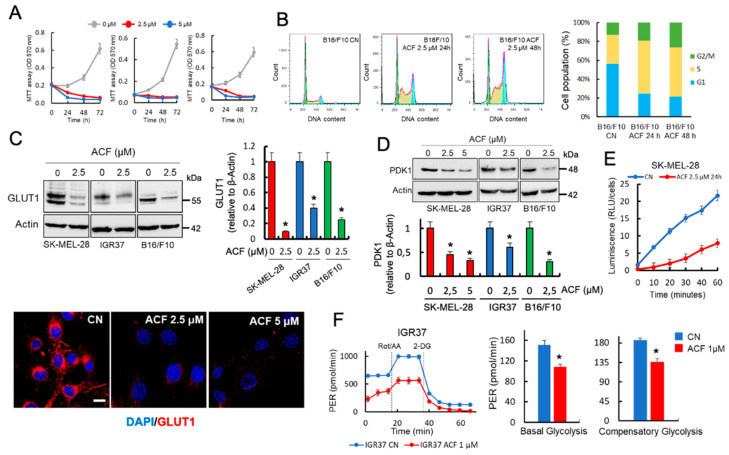
Acriflavine (ACF) restricts glucose availability and suppresses the Warburg effect in melanoma cells. (**A**) Effects of ACF on the growth of indicated melanoma cells. Viability was determined by the MTT assay. The number of surviving cells is directly proportional to the level of the formazan product created and the color can then be quantified at 570 nm. The data values represent the mean from two independent experiments performed in triplicate. (**B**) Cell cycle assays were performed using flow cytometry of B16/F10 cells following ACF indicated treatments. Assays were performed in triplicate, and differences in the cell cycle populations were found to be statistically significant (*p* < 0.05) when treated cells were compared with control cells (CN). (**C**) Western blot (upper panels) and confocal microscopy (63X magnification) (lower panels) showing the expression of GLUT1 in indicated melanoma cells subjected to ACF treatments. The results are representative of three independent experiments. Scale bar, 27 μM. GLUT1 protein expression (histogram) was estimated by integrated optical density (IOD) in western blots after normalization to the β-actin IOD. * *p* < 0.05 when compared with ACF-untreated controls. (**D**) The total levels of PDK1 was examined in indicated melanoma cells using western blot analysis following the indicated ACF treatments. The IOD values (histogram) represent the mean from two experiments performed in triplicate. * *p* < 0.05 when compared with ACF-untreated control experiments. (**E**) Results of the Glucose Uptake-Glo Assay when SK-MEL-28 melanoma cells were treated with ACF. The values represent the mean from two experiments performed in triplicate and the reduction on glucose uptake after ACF was statistically significant at all-time tested (*p* < 0.05). (**F**) Glycolytic proton efflux rate (glycoPER) comparing untreated and ACF-treated IGR37 melanoma cells. The histograms represent individual parameters for basal glycolysis and compensatory glycolysis. IGR37 cells were treated with 1 μM ACF for 24 h and then incubated for 1 h in XF base medium. Each data point represents an ECAR measurement. Data are expressed as means ± SD, n = 5 technical replicates. The graphs are representative of three biological replicates. P values for significant differences (Student’s *t*-test) are summarized by one asterisk (* *p* < 0.001) and groups are compared to ACF-untreated samples.

Cancer cells exhibit altered metabolism with glucose being used as the primary energy source. In melanoma, enhanced glucose usage is favored through the hyper-activated MAPK pathway, which suppresses OXPHOS and stimulates glycolysis [15]. Here, we observed that ACF blocked cell growth in melanoma cells that had been cultured in the presence of physiological fasting concentrations of glucose in human blood (5 mM of glucose) (Figure 1A). Analysis of individual phases of the cell cycle indicated that ACF induces a substantial increase in the number of cells in the S/G2-phase and a decrease in the proportion of cells in the G1-phase in both mouse (B16/F10) and human (SK-MEL-28, IGR37) melanoma cell lines (Figure 1B and Figure S1). Since melanoma cells are dependent on high glucose levels for efficient growth [16] and ACF has been identified as a drug that perturbs intracellular glucose transport [17], we next examined the effect of ACF on the expression of several proteins involved in the transport and usage of glucose in mammalian cells. Interestingly, by western blot and confocal microscopy, we found that ACF induced a dose-dependent decrease in the total amount of GLUT1 in several melanoma cell lines (Figure 1C). In addition, we also observed that ACF induced a significant decrease in the expression of pyruvate dehydrogenase kinase 1 (PDK1), an enzyme that inactivates the TCA cycle enzyme, pyruvate dehydrogenase (PDH), which converts pyruvate to acetyl-CoA (Figure 1D). Therefore, the decrease in GLUT1 and PDK1 activity, two well-recognized HIF-1α-regulated genes, could have a major impact on the transport and usage of glucose in melanoma cells. To validate this hypothesis, we used a non-radioactive assay, based on the detection of 2-deoxyglucose-6-phosphate (2DG6P), to determine the effects of ACF on the glucose uptake of melanoma cells growing under normoxia. As shown in Figure 1E, SK-MEL-28 melanoma cells treated with 2.5 µM ACF during 24 h showed dramatic changes in glucose uptake when compared with untreated control cells.

In addition to increasing energetic stress, glucose deprivation produces the selective death of cancer cells, but not normal cells. It is widely accepted that the main cause of this selective cell death is a reduction in the intracellular antioxidant power of cancer cells, since glucose deprivation decreases the rate of NADPH production from the pentose phosphate cycle and glucose-derived one-carbon metabolism. The reduction in antioxidant capacity then leads to an increase in intracellular reactive oxygen species (ROS) [18]. Since HIF-1α promotes glycolysis while repressing mitochondrial activity [19], we next asked whether ACF could affect glycolytic metabolism. Using a Seahorse platform, we observed in Figure 1F that ACF suppressed the Warburg effect in IGR37 melanoma cells, significantly decreasing basal and compensatory glycolysis under aerobic conditions (see also Figure S2 for other cell lines).

### 2.2. Acriflavine Differentially Modulates HIF-1α-Dependent Pathways in Melanoma Under Normoxic Conditions

**Figure 2:**
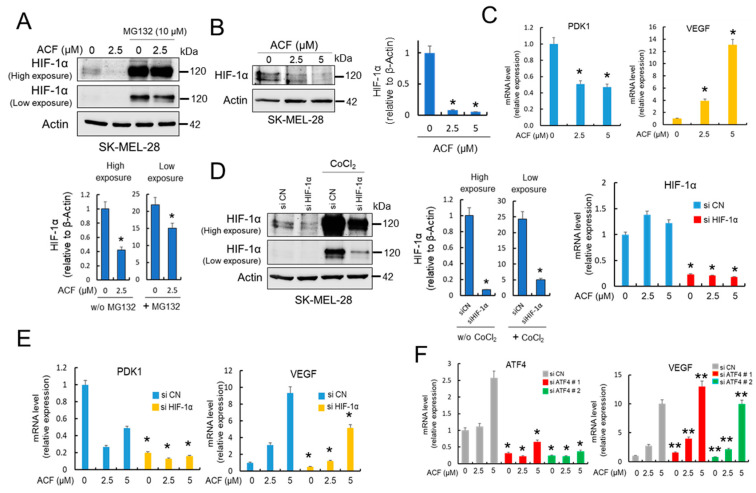
ACF differentially modulates PDK1 and VEGF transcription in melanoma cells. (**A**) Western blot experiments for the effect of ACF on HIF-1α expression in the absence or the presence of MG132. SK-MEL-28 cells were treated for 12 h with ACF alone or simultaneously co-treated with ACF and MG132. High and low exposure make reference to the exposure time during development of the chemiluminescent signal. HIF-1α expression in the absence of MG132 (w/o MG132) was quantified on high exposure membranes, while that of HIF-1α expression in the presence of MG132 was evaluated on low exposure membranes to avoid overexposure (histograms). In both cases, relative HIF-1α content in the ACF-treated samples was compared with untreated controls in membranes developed under the respective exposure conditions (* *p* < 0.05). (**B**) Dose-dependent effect of ACF (24 h) on HIF-1α and IOD quantification (histogram; * *p* < 0.05). (**C**) Semiquantitative determination of PDK1 and VEGF mRNAs in SK-MEL-28 cells. Relative levels of mRNA (with respect to β-actin) in ACF-treated samples (24 h) were compared to the expression levels in untreated controls (* *p* < 0.05). (**D**) Effective silencing of HIF-1α was determined by both western blot experiments and mRNA determinations in SK-MEL-28 melanoma cells. To visualize the silencing of HIF-1α in western blots, the protein was stabilized with CoCl_2_ (200 µM). Images were obtained under high and low exposure as indicated in Figure 2A and quantified in indicated membranes (histograms) in the absence (w/o CoCl_2_) or the presence of CoCl_2_ (* *p* < 0.05). HIF-1α-mRNA in silenced samples were compared with their respective ACF treatments (24 h) in siControls (siCN) samples and differences were found statistically significant (* *p* < 0.05). (**E**) Semiquantitative determination of PDK1 and VEGF mRNAs in HIF-1α-silenced SK-MEL-28 cells. HIF-1α-mRNA in silenced samples were compared with their respective treatments in siCN samples (* *p* < 0.05). Although VEGF-mRNA decrease in siHIF-1α cells was found to be statistically significant with respect to siCNs, silencing of HIF-1α did not completely abolish ACF-dependent induction of VEGF. Cells were treated with ACF for 24 h. (**F**) Effective silencing of ATF4 with two different siRNAs (left panel) did not influence VEGF-mRNA expression in SK-MEL-28 cells. * *p* < 0.05 and ** not significant when compared with siATF4 samples with their respective treatments in siCN samples. When indicated, cells were treated with ACF for 24 h.

ACF has been identified as an efficient inhibitor of HIF-1α dimerization and consequently has potent inhibitory effects on tumor growth and vascularization [14]. However, since this transcription factor operates primarily under hypoxic conditions, most studies aimed at testing the efficacy of this drug in different cancer cell models have been carried out under oxygen-limiting conditions [14]. Here, and in addition to its proposed inhibitory activity, we also observed that ACF modulated the expression of HIF-1α under normoxic conditions (Figure 2AB). These results, together with the effect of MG132 treatment (Figure 2A) make it tempting to speculate that ACF could modulate the transcriptional activity of HIF-1α in normoxia when melanoma cells are growing under glucose-limiting conditions. To investigate this possibility, we analyzed the mRNA expression levels of PDK1 and VEGF, two well-known targets of HIF-1α [20,21]. Interestingly, as we observed for their protein levels (Figure 1D), ACF decreased PDK1-mRNA levels, but had an opposite effect on the expression of VEFG-mRNA (Figure 2C). To address the effect of ACF on the expression levels of PDK1 and VEGF in melanoma cells, we then conducted knock-down experiments of HIF-1α. Although effective silencing of HIF-1α (Figure 2D) clearly influenced the levels of PDK1-mRNA and eliminated the dose-dependent effect of ACF (Figure 2E), the increased expression of VEGF after ACF was diminished but not abolished after HIF-1α silencing (Figure 2E). Altogether, these results indicate that ACF differentially modulates HIF-1α-dependent pathways in melanoma, and suggests that under normoxic conditions VEGF expression could be regulated by HIF-1α-independent mechanisms. Since VEGF has been shown to be coordinately regulated by both HIF-1α and activating transcription factor 4 (ATF4) in normoxia [20,22], we next silenced ATF4 in cells treated with different concentrations of ACF (Figure 2F). The results showed that silencing ATF4 did not suppress ACF-dependent induction of VEGF, indicating that in normoxia, VEGF mRNA expression can be regulated by different mechanisms.

### 2.3. Acriflavine Decreases MITF Expression in Melanoma Cells in an ATF4-independent Manner

**Figure 3:**
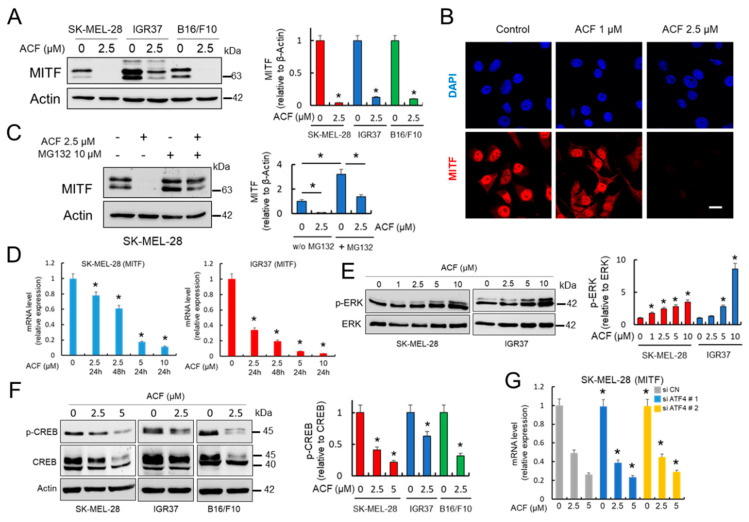
ACF decreases MITF expression in melanoma cells. (**A**) Effect of ACF (24 h) on MITF expression in melanoma cells analyzed by western blot. IOD quantification is shown (histogram; * *p* < 0.05 when compared with untreated controls). (**B**) Confocal microscopy analysis (63X magnification) of MITF in SK-MEL-28 melanoma cells under indicated conditions (cells were treated with ACF for 24 h). Bars, 27 μM. (**C**) Western blot experiments for the effect of ACF on MITF expression in the absence or the presence of MG132. SK-MEL-28 cells were treated for 12 h with ACF alone or simultaneously co-treated with ACF and MG132. * *p* < 0.05 when comparing indicated data groups. (**D**) qRT-PCR analysis of MITF mRNA in indicated melanoma cells before and after ACF treatments (24 h). Relative mRNA expression in treated cells was normalized with respect to untreated cells. * *p* < 0.05. (**E**) Effect of ACF (24 h) on the phosphorylation of ERK1/2 (p-ERK) in melanoma cells analyzed by western blot. Specific antibodies recognized the diphosphorylated forms of ERK1/2 (Thr183 and Tyr185 based in ERK2 nomenclature). Constitutive total ERK was used as a reference for p-ERK expression. IOD quantification is shown (histogram; * *p* < 0.05 when compared with untreated controls). (**F**) Effect of ACF (24 h) on Ser133 phosphorylation in CREB (p-CREB). Western blot of total CREB showed two clear bands at 40 and 45 kDa, corresponding to the upper band to phosphorylated CREB. IOD quantification is shown (histogram; * *p* < 0.05 when compared with untreated controls). (**G**) Effective silencing of ATF4 with two different siRNAs (Figure 2F) did not influence MITF-mRNA expression in SK-MEL-28 cells. * not significant when compared to siATF4 samples with their respective treatments in siCN samples. When indicated, cells were treated with ACF for 24 h.

Accumulating evidence suggests that a key determinant of melanoma phenotype is the expression and activity of the microphthalmia-associated transcription factor (MITF) [23]. MITF represents a major coordinator of melanoma cell biology. It promotes survival, differentiation, and proliferation, and plays a critical role in regulating melanoma metabolism [24,25,26]. Therefore, here, we evaluated the expression of MITF in the presence of ACF. As shown in Figure 3A,B, MITF protein levels were downregulated in a dose dependent manner by ACF in several melanoma cell lines as demonstrated by western blot and confocal microscopy. MG132, a specific proteasome inhibitor, partially recovered the MITF protein in the presence of ACF, potentially indicating that ACF decreased MITF protein stability in melanoma cells (Figure 3C). However, as demonstrated by qRT-PCR, ACF also regulates *MITF* transcription, since the amount of MITF-mRNA decreased dramatically after ACF treatment in a dose-dependent manner (Figure 3D). To decipher the effects of ACF on MITF expression, we next analyzed the phosphorylated forms of ERK1/2 and CREB in melanoma cells (Figure 3E,F). While p-CREB has been identified as a transcription factor that binds and activates the *MITF* promoter via the cyclic adenosine monophosphate (cAMP) response element, ERK1/2 activation has been reported to drive MITF phosphorylation and degradation. The results indicate that ACF moderately increased ERK phosphorylation/activation, but also reduced CREB phosphorylation.

ATF4 has been identified as a potent repressor of MITF mRNA expression [25], and glucose restriction promotes ROS-dependent induction of ATF4, which in turn can suppress MITF mRNA expression by competing with p-CREB binding to the CRE site of the *MITF* promoter [16]. Since ACF decreased glucose availability and affected the HIF-1α pathway under normoxic conditions, we hypothesized that the induction of an ER stress response pathway could be responsible for the ACF-dependent suppression of MITF in melanoma cells. To determine if ATF4 was involved in the decreased expression of MITF in the presence of ACF, we next analyzed MITF mRNA expression in ATF4 silenced cells. As shown in Figure 3G, ACF can induce a decrease in MITF expression in melanoma cells even in the absence of ATF4. Collectively, these results indicate that ACF is likely to decrease MITF levels in melanoma cells by modulating the phosphorylation of ERK1/2 and CREB in a mechanism independent of ATF4.

### 2.4. Acriflavine Activates Endoplasmic Reticulum Stress Pathways While Compromises ATF4 Stability in Melanoma Cells

**Figure 4:**
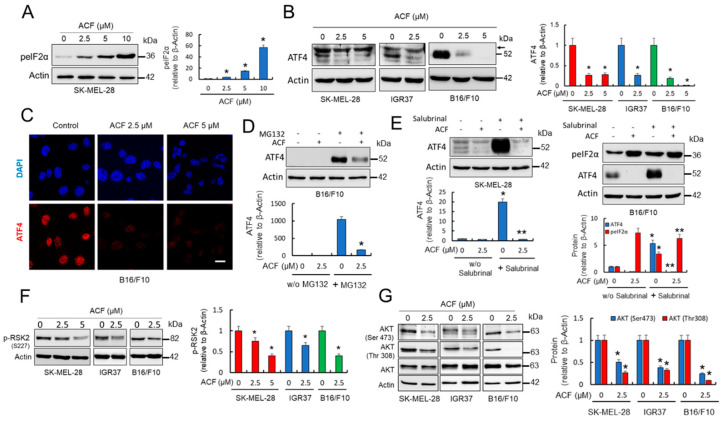
ACF compromises ATF4 protein stability in melanoma cells. (**A**) Effect of ACF (24 h) on p-eIF2α expression in SK-MEL-28 melanoma cells analyzed by western blot. IOD quantification is shown (histogram; * *p* < 0.05 when compared with untreated control). (**B**) Western blot analysis of ATF4 in melanoma cells under indicated conditions (cells were treated with ACF for 24 h). Histogram represents ATF4 protein levels (detected as a band at 52 kDa) estimated by IOD in western blots after normalization to the β-actin IOD. The values represent the mean from two experiments performed in triplicate. * *p* < 0.05 when compared with ACF-untreated control experiments. Arrow indicates a nonspecific band observed in human samples at 58 kDa. (**C**) Confocal microscopy analysis (63X magnification) of ATF4 in control B16/F10 cells and those subjected to indicated ACF (24 h) treatments (bars, 27 μM). (**D**) Western blot experiments for the effect of ACF (2.5 µM) on ATF4 expression in the absence or the presence of MG132 (10 µM). SK-MEL-28 cells were treated for 12 h with ACF alone or simultaneously co-treated with ACF and MG132. MG132 significantly increased ATF4 in ACF-treated cells (* *p* < 0.05). (**E**) Western blot experiments for the effect of ACF (2.5 µM) on ATF4 expression in the absence or the presence of salubrinal (20 µM). Melanoma cells were treated during 24 h with ACF alone or simultaneously co-treated with ACF and salubrinal. As observed, ACF impedes ATF4 stabilization under forced stabilization of p-eIF2α * *p* < 0.05 and ** not significant when compared salubrinal treatments with their respective treatments without salubrinal (w/o salubrinal). (**F**) Effect of ACF (24 h) on the phosphorylation of RSK2 (p-RSK2) in melanoma cells analyzed by western blot. p-RSK2 (histogram) was estimated by integrated optical density (IOD) in western blots after normalization to the β-actin IOD. The values represent the mean from two experiments performed in triplicate. * *p* < 0.05 when compared with the ACF-untreated control experiments. (**G**) AKT phosphorylation was examined in melanoma cell extracts and compared with the expression levels of total AKT and β-actin. IOD quantification is shown (histogram; * *p* < 0.05 when compared with their respective untreated controls).

Nutrient deprivation in tumor cells triggers endoplasmic reticulum (ER) stress with the subsequent activation of ATF4 [27,28]. Whether ACF induces ER stress and activates ATF4-dependent pathways was further investigated. That ACF activates an ER stress response in melanoma cells was evident from the analysis of the phosphorylation of eIF2α (Figure 4A), a stress-induced factor that blocks general protein synthesis and activates the translation of several stress-response and pro-apoptotic proteins. Consistent with induction of ER-stress, ACF significantly increased p-eIF2α in a dose-dependent manner. We next examined the levels of ATF4 in the controls and melanoma cells subjected to ACF treatments, since in contrast to global translation, which is downregulated by eIF2α phosphorylation, ATF4 translation is increased. Although ATF4 was detected in untreated cells, as observed in melanoma cells growing under low glucose conditions [16], unexpectedly, we found that ACF consistently induced a decrease in ATF4 protein in melanoma cell lines (Figure 4B,C). Since p-eIF2α reduces general translation initiation while facilitating the preferential translation of select transcripts such as that encoding ATF4, the results seem to indicate that ACF may lead to a destabilization of ATF4-protein in melanoma cells. In agreement, we observed that the ACF-dependent decrease of ATF4 protein in SK-MEL-28 cells was not accompanied by a concomitant decrease in ATF4-mRNA levels; instead, ACF increased the levels of ATF4-mRNA in this melanoma cell line (Figure 2E). Although we observed a high recovery of ATF4 after co-treatment of melanoma cells with ACF and MG132 (Figure 4D), suggesting ATF4 protein stability was reduced by ACF, MG132, per se, is an activator of the unfolded protein response (UPR), making interpretation of this experiment difficult. Interestingly, experiments designed to increase p-eIF2α with salubrinal, a specific inhibitor of eIF2α phosphatase, also seem to indicate that ACF induced destabilization of ATF4 protein in melanoma cells (Figure 4E).

In trying to explain the observed destabilization of ATF4 protein, we concentrated on RSK2 as phosphorylation of ATF4 by RSK2 stabilizes ATF4 and prevents its degradation by the proteasome [29]. The results in Figure 4F revealed that ACF significantly reduced phosphorylated (activated) RSK2 in melanoma cells. This observation might also explain the mechanism of action by which ACF reduces MITF expression in melanoma, since the RSK2 pathway also converges on CREB phosphorylation [30]. Interestingly, we also observed that ACF treatment promoted the inhibition of AKT phosphorylation (Figure 4G). Since phosphorylation of both AKT and RSK2 is governed by phosphoinositide-dependent kinase-1 (PDPK1), the results suggest that ACF blocks the PI3K pathway upstream of AKT and RSK2 phosphorylation. As the PDPK1-AKT pathway, in addition to activating the transcription of HIF-1α [31,32], also promotes cell surface expression of GLUT1, inhibition of AKT phosphorylation by ACF might have a major impact on glucose metabolism in melanoma cells.

### 2.5. Acriflavine Induces Melanoma Cell Death under Normoxic Conditions

**Figure 5:**
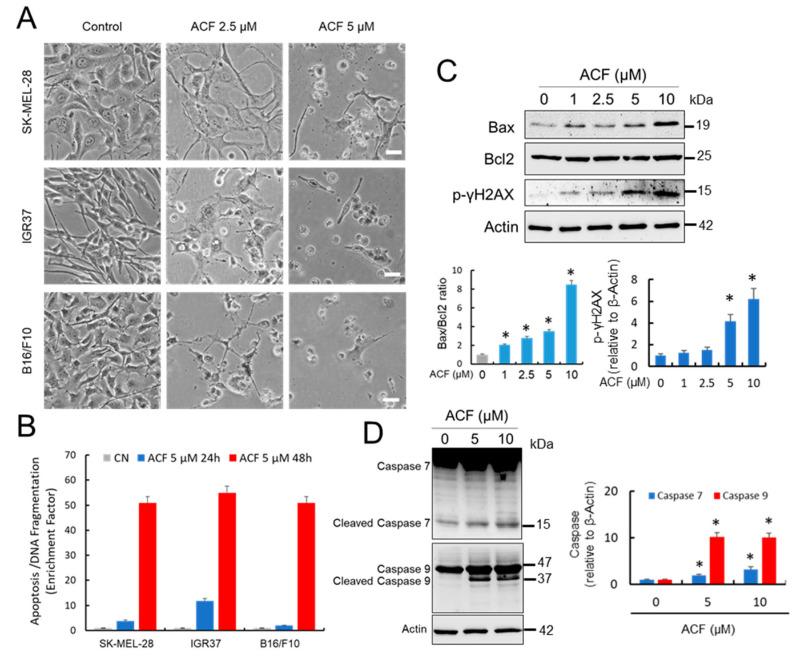
ACF induces apoptotic cell death in melanoma cells. (**A**) Morphological aspect of untreated melanoma cells compared with those subjected to 2-days of treatment with indicated concentrations of ACF (bars, 100 μM). 40× magnification (**B**) Apoptosis determination at different ACF concentrations in indicated melanoma cells after 24 and 48 h of treatment. Data were obtained in triplicate in two independent experiments. Differences in apoptosis in ACF-treated cells were significant with respect to untreated controls for each drug concentration and at any time (*p* < 0.05). (**C**) Western blots showing the effect of ACF on Bax, Bcl2, and p-γH2AX proteins. SK-MEL-28 cells were treated with different concentrations of ACF for two days. The ratios between Bax and Bcl2 and relative p-γH2AX are presented in the histograms (* *p* < 0.05 when compared with the untreated control). (**D**) Western blot analysis of caspase 7 and caspase 9 in the control SK-MEL-28 cells and those treated with indicated doses of ACF. IOD quantification is shown (histogram; * *p* < 0.05 when compared with their respective untreated controls).

Cancer cells require a continuous supply of nutrients to maintain cell division [27]. In this context, the adaptation of these cancer cells to a limited availability of nutrients is facilitated by ATF4, one of the master regulators of the cellular stress response. Since ACF, by inhibiting HIF-1α-regulated pathways in normoxia, can reduce glucose availability and induce ER stress while suppressing ATF4 protective pathways, this drug could represent an efficient treatment against melanoma. We therefore analyzed the pro-apoptotic activity of ACF on several melanoma cell lines (Figure 5A,B). Indeed, we observed that blockage of cell growth (Figure 1A) was also accompanied by a substantial increase in melanoma apoptosis together with visible DNA damage, as reflected by an ACF-induced increase in p-γH2AX. In addition, melanoma cells treated with ACF exhibited a significant increase in Bax/Bcl2 ratio, a predictive marker for therapy response (Figure 5C). Clear activation of caspase 7 and caspase 9 after ACF treatment (Figure 5D) also indicated that this drug induced cell death in melanoma cells under normoxic conditions.

## 3. Discussion

HIF-1 plays a predominant role in the response of cells to hypoxia, a microenvironmental condition that is particularly relevant during tumor development. However, whether HIF-1α-related pathways are operative under normoxia and whether cancer cells originating from different tissues are dependent of HIF-1α under non-hypoxic stress conditions are questions that remain to be elucidated [6]. In this respect, the use of HIF-1α inhibitors such as ACF could constitute a useful tool to identify HIF-1α pathways operating under normoxia that are essential for survival in response to cellular stresses such as nutrient deprivation or ROS production, among others. Here, we analyzed the effect of ACF, a HIF-1α inhibitor, on melanoma cells maintained at 5 mM glucose. Interestingly, melanoma cells grown at this glucose concentration, corresponding to fasting physiological concentrations, exhibit a high rate of ROS production, which leads to the activation of ATF4 [16]. The results of this study clearly indicated that competition between ATF4 and CREB for their localization at the *MITF* promoter resulted in repression of MITF expression. We therefore used this cellular model to elucidate the role of HIF-1α in cells subjected to oxidative metabolic stress under normoxia.

Another important issue is to understand whether the HIF-1α metabolic controlled pathways are different when they operate under normoxia or hypoxia. Although VEGF has been considered as a paradigm of genes controlled by HIF-1α under hypoxia, here, we observed that HIF-1α is not completely required for VEGF expression under normoxic conditions. Interestingly, it has been proposed that the interaction between ER stress and hypoxia response pathways can potentiate HIF-1 transcriptional activity at the *VEGF* gene [20], and oxidative stressors that induce ATF4-dependent VEGF mRNA transcription [22]. However, our results did not indicate that ATF4 could be responsible for VEGF mRNA induction after treatment with ACF. That HIF-1α-regulated pathways may differ when they operate under hypoxic or normoxic conditions could also be deduced from observations, suggesting that HIF-1α could regulate MITF transcriptional activity. Thus, a previous report has suggested that the pro-angiogenic response of melanoma to low levels of oxygen depends at least in part on HIF-1α mediated downregulation of MITF [33]. However, here, we did not find that HIF-1α downregulated MITF expression under normoxia; in fact, we observed that inhibition of HIF-1α by ACF resulted in a significant decrease in MITF expression. Since the repression of MITF by HIF-1α was shown to be controlled by the hypoxia-dependent recruitment of DEC1 to the *M-MITF* promoter, it is reasonable to speculate that hypoxia-dependent stabilization of factors necessary for HIF-1 activity may differentially regulate HIF-1 dependent pathways under normoxic or hypoxic conditions.

**Figure 6:**
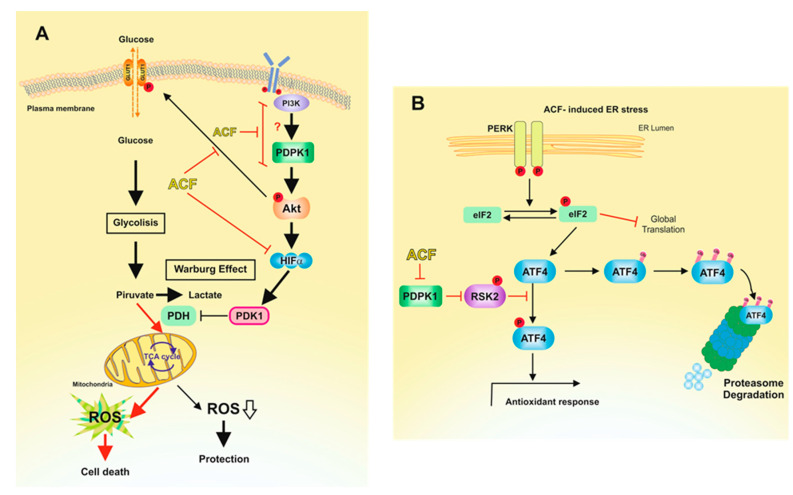
Proposed mechanisms for the action of ACF on melanoma cells under normoxic conditions. (**A**) This picture reproduces the adaptation of melanoma cells to physiological concentrations of glucose. Restriction of glucose availability to physiological concentrations induces the production of ROS [16], which activate HIF-1α [46]. Activated HIF-1α induces glycolysis upregulation in cancer cells, a phenomenon known as the Warburg effect [47]. Thus, by increasing the conversion of pyruvate to lactate, the Warburg effect reduces ROS production by the mitochondrial OXPHOS. Activated AKT pathway contributed to glucose transport through GLUT1 plasmatic membrane translocation and activation of HIF-1α. ACF, by inhibiting HIF-1α, impedes PDK1 transcription, resulting in enhanced ROS production. In this metabolic scenario, ACF blocks the PI3K/PDPK1 pathway, resulting in impaired phosphorylation of AKT. Consistently with our results (Figure 2A), inhibition of AKT phosphorylation by ACF would also result in reduced expression of HIF-1α [31] under normoxic conditions. Red arrows indicate favored pathways in the presence of ACF. (**B**) Increased ROS levels induces ER stress, leading to the UPR [48]. Although ACF induces the phosphorylation of eIF2α, this is not traduced in elevated expression of ATF4. Since protein stability of ATF4 is dependent of an operative PDPK1/RSK2 pathway [29], ACF induces the destabilization of ATF4 and, therefore, the inactivation of ATF4-adaptative pathways.

Collectively, the results presented indicate that ACF, by inhibiting the transcriptional activity of HIF-1 in normoxia, negatively regulates PDK1 in melanoma cells, which could explain many of the effects observed after ACF treatment (Figure 6). In recent years, PDK1 has been the focus of many studies aimed at blocking glucose metabolism in tumor cells [34]. Since PDK1 inhibits PDH, its action would facilitate the Warburg effect by reducing the levels of pyruvate entering the tricarboxylic acid cycle and affecting the rates of OXPHOS [35] (Figure 6A). In this scenario, HIF-1α upregulates PDK1 and increases glucose uptake by GLUT1 transporters. Therefore, by inhibiting HIF-1α, ACF suppresses the Warburg effect and impedes the adaptation of melanoma cells to oxidative stress (Figure 6A). In addition, several oncogenes have been identified as key players associated with the HIF-1α-mediated Warburg [36]. For instance, the signaling pathway composed of PI3K, AKT, and mTOR is associated with the upregulation of HIF-1α in normoxic conditions [32]. In this sense, in addition to activating HIF-1α dependent transcription of GLUT1 transporters, an active AKT pathway may also facilitate GLUT1 translocation to the plasma membrane [37,38]. Although there are reports suggesting that AKT activation can protect cells under glucose deprivation, regulation of this oncogenic pathway under glucose restriction has been shown to be sophisticated and specific to different cancer cells and backgrounds [39]. Thus, for example, AKT is activated in glioblastoma cells [40], but inhibited in ovarian cancer cells [41]. Here, we observed that the restriction of glucose availability to physiological concentrations favors an operative AKT pathway in melanoma cells; however, inhibition of the Warburg effect by ACF resulted in the inhibition of AKT phosphorylation, probably due to an excessive production of ROS levels that render cells closer to the threshold of ROS lethality (Figure 6A).

In addition to disturbing glucose metabolism in melanoma cells, the inhibition of the PI3K/PDPK1 pathway by ACF may also have important consequences for the pathobiology of these cells. On one hand, inactivation of AKT by ACF may be important to maintain the stability of MITF in melanoma cells. GSK3β phosphorylates MITF at 3 C-terminal phosphorylation sites, targeting MITF for proteasomal degradation [42]; therefore, inhibition of AKT by ACF would result in decreased MITF protein stability. On the other hand, we observed that ACF reduced the phosphorylation of RSK2, a kinase that is controlled through its phosphorylation by PDPK1 at Ser227 [43]. RSK2 might modulate ATF4-related pathways. Melanoma cells growing under limiting glucose concentration activate rescue routes to condition themselves to the nutritional conditions of the environment. Among these pathways, limiting glucose availability results in the expression of the transcription factor ATF4, which is a known mediator of stress pathways including hypoxia/anoxia, nutrient deprivation, and endoplasmic reticulum stress [16]. Since the stabilization of the ATF4 protein is dependent on an active PDPK1/RSK2 pathway [29], inhibition of RSK2-mediated ATF4 phosphorylation by ACF may compromise the pro-survival activity of ATF4 (Figure 6B). In fact, we observed that ACF dramatically decreased the protein levels of ATF4 in melanoma cells. These results agree with other observations that suggest that ACF inhibits acquired drug-resistance by blocking the epithelial-to-mesenchymal transition and the UPR in pancreatic cancer cells [44]. However, it is important to note that our data were generated in cell lines in culture and whether the pathways identified operate in vivo requires validation in an in vivo model. Nevertheless, the observation that ACF indirectly targets ATF4 in melanoma cells could be interesting from a therapeutic point of view [45]. Since ATF4 is a poor drug target, but plays important functions in cancer progression and resistance to therapy, the action of a small molecule such as ACF that indirectly targets ATF4 for proteasomal degradation could open new avenues for the treatment of melanoma. Since ACF treatment clearly induced melanoma cell death in in vitro models, we are now interested in testing the action of this compound in pre-clinical models of melanoma. From these future results, we will be able to draw conclusions indicating whether ACF could be transferred to a clinical setting.

## 4. Materials and Methods

### 4.1. Chemicals

ACF, Thiazolyl Blue Tetrazolium Bromide (MTT), CoCl_2_, salubrinal, and MG132 were obtained from Merck (Madrid, Spain). Eagle’s Minimum Essential Medium (EMEM), fetal bovine serum (FBS), trypsin-EDTA, penicillin, and streptomycin were purchased from Gibco (Thermo-Fisher, Barcelona, Spain). Antibodies used in this study are indicated in the Supplementary Materials (Table S1).

### 4.2. Cell Cultures

Authentication of the SK-MEL-28, IGR37 human melanoma cells, and mouse B16/F10 melanoma cells was performed using STR profiling. All cell lines were mycoplasma free and cultured at 37 °C and 5% CO_2_ in EMEM without phenol red supplemented with 10% FBS, 2 mM glutamine, and 1% penicillin and streptomycin.

### 4.3. Cell Viability Assays

The cell viability was determined using the MTT assay. Cells (1000–2000) were plated in 96-well flat-bottom plates. The next day, once cells were attached, they were treated with ACF and maintained in culture for the indicated times. MTT reagent was dissolved in culture medium (5 mg/mL), added to the cells to a final concentration of 1 mg per well, and incubated for two hours. Formazan crystals were dissolved using DMSO and absorbance was measured on a microplate reader (Fluostar-Omega, BMG Labtech., Ortenberg, Germany) at 540 nm using a 650 nm wavelength measured as background reference.

### 4.4. Cell Cycle Analysis and Flow cytometry

Experiments were performed in a 6-well plate format. Cells were treated with ACF 2.5 μM for 24 h and 48 h. After treatment, cells were washed in PBS, fixed with 70% ethanol for 1 h at 4 °C and incubated for 30 min at 37 °C in propidium iodide staining solution (50 μg/mL RNase A, 50 μg/mL propidium iodide, and 0.05% Triton X-100 in PBS). DNA fluorescence was measured with a FACSort cytometer (Becton-Dickinson, Franklin Lakes, NJ, USA) and cell cycle phases evaluated using FlowJo software (FlowJo, Ashland, OR, USA).

### 4.5. Apoptosis

The effect of ACF on apoptosis induction of melanoma cells was determined using an ELISA assay (Cell Death Detection ELISA PLUS, Roche Diagnostics, Barcelona, Spain). This is a photometric enzyme immunoassay that quantifies the presence of histone associated DNA fragments (nucleosomes) present in the cytoplasm of apoptotic cells using anti histone-biotin and anti-DNA-peroxidase conjugated antibodies. The assay was developed in 96-well flat-bottom plates and cells treated with ACF 5 μM for 24 h and 48 h. Next, cells were lysed and plates were centrifuged. The load of nucleosomes in the cytoplasm of cells was analyzed by measuring the absorbance at 405 nm using ABTS as a substrate in a microplate reader (Fluostar-Omega, BMG Labtech., Ortenberg, Germany). Apoptosis fold was calculated relative to untreated cells considering the number of cells.

### 4.6. Western Blotting

Protein samples were obtained directly from cell lysis in Laemmli buffer or extracted using lysis buffer (50 mM TRIS-HCl pH 8.0, 180 mM NaCl, 1% NP-40) followed by dilution in Laemmli buffer. After denaturation at 95 °C for 5 min, proteins were resolved by electrophoresis SDS-PAGE using 10% acrylamide gels. After separation, proteins were transferred to nitrocellulose membranes (Merck) in a Bio-Rad transfer system and membranes were blocked in 5% nonfat milk and incubated overnight with the indicated primary antibodies diluted in blocking solution. After washing, membranes were incubated with HRP-conjugated secondary antibodies (anti-mouse, anti-rabbit, or anti-rat IgG) diluted 1:10,000 in blocking solution for one hour. Following extensive washing, the signal was detected by incubation of the membranes with an ECL detection kit (WesternBright Quantum, Advansta, San Jose, CA, USA). Bands were visualized and images were recorded with a Bio-Rad ChemiDoc scanning densitometer (Bio-Rad Laboratories, Hercules, CA, USA). The original western blotting figures can be found in Figure S3.

### 4.7. Confocal Microscopy

Cells were grown on glass coverslips and fixed and permeabilized using 100% methanol for 5 min and washed with PBS. Coverslips were blocked with 5% BSA in PBS for 20 min and probed with the indicated primary antibodies (ATF4, MITF, and GLUT1) at 4 °C. After washing the excess of antibody solution with PBS, proteins were detected with Alexa Fluor Dyes (Alexa Fluor 633 rabbit anti-mouse IgG (H+L) and Alexa Fluor 633 goat anti-rabbit IgG (H+L) both from Thermo Fisher Scientific) and imaged using a Leica TCS 4D confocal microscope (Wetzlar, Germany).

### 4.8. Glucose Uptake Assay

The non-radioactive, plated-based bioluminescent assay Glucose Uptake-Glo^TM^ (Promega, Madison, WI, USA) based on the detection of 2-deoxyglucose-6-phosphate was used for measuring glucose uptake in the melanoma cells. Reactions were developed in 96 well-plates following the manufacturer’s recommended protocol. Glucose uptake was determined over time for the control and ACF-treated cells measuring the luminescent signal produced with a multiplate luminescence recorder (Fluostar-Omega).

### 4.9. Real Time Quantitative RT-PCR

Extraction of RNA from melanoma cells was performed using the NZY total RNA Isolation Kit (Nzytech, Lisboa, Portugal) and cDNA prepared using the NZY First-Strand cDNA Synthesis Kit (Nzytech). Primers used in this study are included in the Supplementary Materials (Table S2). Primers were designed using Primer Blast software (NCBI) and obtained from Thermo-Fisher. mRNA expression levels were evaluated by qRT-PCR (QuantStudio 5 Real-Time PCR System, Applied Biosystems, Foster City, CA, USA) using the SYBR green master mix (Applied Biosystems) and normalized to β-actin.

### 4.10. Metabolic Assay: Glycolytic Rate Assay

Glycolytic proton efflux rate (GlycoPER) was measured using the Seahorse XFe96 extracellular flux analyzer (Seahorse Bioscience, North Billerica, MA, USA). Cells were seeded in XF96 well plates at a density from 5000 to 15,000 cells per well to reach approximately 75% confluence. Cell were allowed to attach to the bottom of the well at room temperature before going into the incubator at 37 °C in 5% CO_2_ to ensure their homogeneous distribution. The next day, cells were treated with 1 µM ACF for 24 h prior to the assay. One hour before the glycolytic rate analysis, the medium was replaced with fresh Seahorse XF DMEM medium without phenol red supplemented with 1 mM pyruvate, 2 mM glutamine, and 10 mM glucose. ECAR and OCR were both measured in the Seahorse XFe96 analyzer at basal conditions and after the sequential injections of 0.5 µM Rotenone plus Antimycin A (Rot/AA) and 50 mM 2-deoxyglucose (2-DG) (XF Glycolytic Rate Assay Kit). Values were normalized to cell number.

### 4.11. siRNA and Cell Transfection

HIF-1α-siRNA (s6539), ATF4-siRNA [siATF4#1 (s62689) and siATF4#2 (s62691)] and non-silencing siRNA (4390843) were purchased from Ambion (Thermo-Fisher). Cells were seeded on six/well culture dishes to approximately 70% confluence. Transient transfection was performed using Lipofectamine 2000 (Thermo-Fisher) following the manufacturer’s instructions. Cells were incubated with transfection mixtures containing HIF-1α-siRNA, ATF4-siRNA, or non-silencing siRNA for 72 h.

### 4.12. Statistical Analysis

Western blot and confocal microscopy experiments were repeated at least three times. The results of one of the experiments are shown in the figures. For other assays, the mean ± S.D of three determinations carried out in triplicate were calculated. Statistical significance was determined using Mann–Whitney tests for comparisons of means in SPPS statistical software for Microsoft Windows, release 6.0 (Professional Statistic, Chicago, IL, USA). Individual comparisons were made using the Student’s two-tailed, unpaired *t*-tests. Criterion for significance was *p* < 0.05 for all comparisons.

## 5. Conclusions

The incidence of melanomas is increasing and, despite advances in targeted and immunotherapies, the prognosis for many patients with advanced disease is not very promising. Without a doubt, understanding tumor cell oncometabolism may generate new opportunities to generate new, more effective, and safer therapies against epithelial tumors. Here, we describe the mechanism of action of a drug, ACF, which could modulate the metabolism of melanoma cells, independently of the hypoxic conditions of the tumor. Interestingly, by inhibiting the HIF-1α/PDK1 axis, ACF modulates the metabolism of glucose in melanoma cells. In addition, by inhibiting the PI3K/PDPK1 pathway, ACF also blocks important adaptive mechanisms necessary for cell survival under metabolic stress.

## Data Availability

The data presented in this study and not included within the Supplementary Materials are available on request from the corresponding author.

## Supplementary Materials

The following are available online at https://www.mdpi.com/2072-6694/13/1/102/s1, Figure S1. Cell cycle assays of SK-MEL-28 (**A**) and IGR37 (**B**) cells following ACF indicated treatments. Assays were performed as indicated in Figure 1B. Figure S2. Glycolytic proton efflux rate (glycoPER) comparing untreated and ACF-treated melanoma cells. (**A**) SK-MEL-28, (**B**) B16/F10. Assays were performed as indicated in Figure 1F. Figure S3: Uncropped western blotting figures. Table S1. Used primary antibodies. Table S2. The following primers for human genes were used.
