# Supplementary material for: Acriflavine, a Potent Inhibitor of HIF-1α, Disturbs Glucose Metabolism and Suppresses ATF4-Protective Pathways in Melanoma under Non-hypoxic Conditions
Source: Cancers (Basel). 2020 Dec 31;13(1):102. doi: 10.3390/cancers13010102 (PMC7795823; doi:10.3390/cancers13010102)
Supplement: Supplementary file 1 [file cancers-13-00102-s001.pdf]

cancers

Article

Acriflavine, a potent inhibitor of HIF1α, disturbs glucose metabolism and suppresses ATF4-protective pathways in melanoma under non-hypoxic conditions

Román Martí-Díaz, María F. Montenegro, Juan Cabezas-Herrera, Colin R. Goding, José Neptuno Rodríguez-López and Luis Sánchez-del-Campo

Supplementary Material

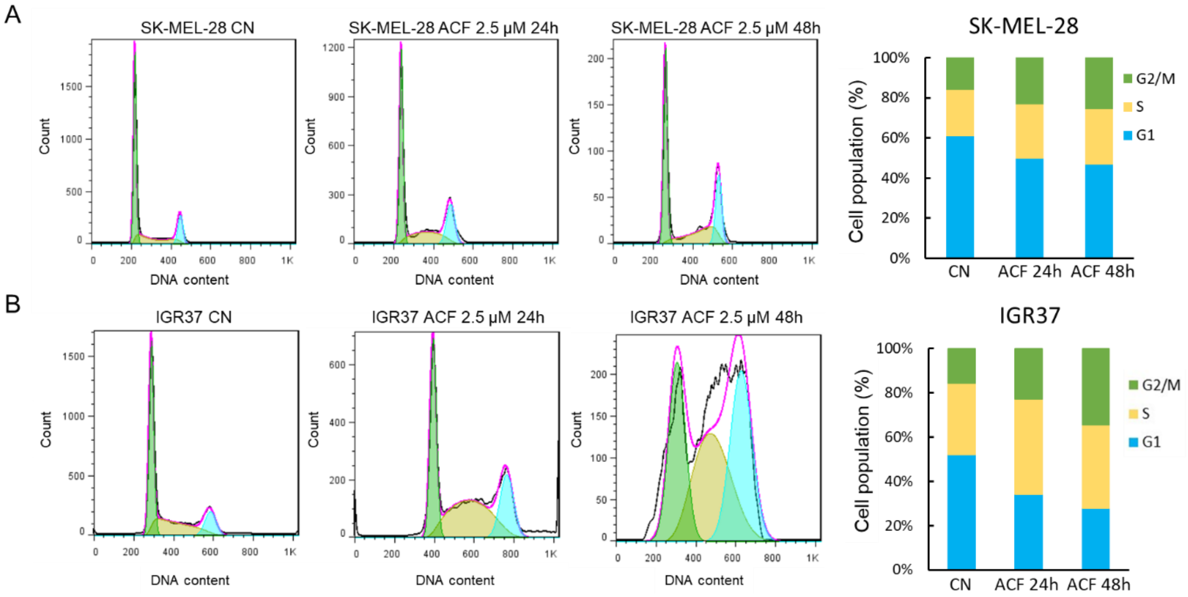

Figure S1. Cell cycle assays of SK-MEL-28 (A) and IGR37 (B) cells following ACF indicated treatments. Assays were performed as indicated in Figure 1B.

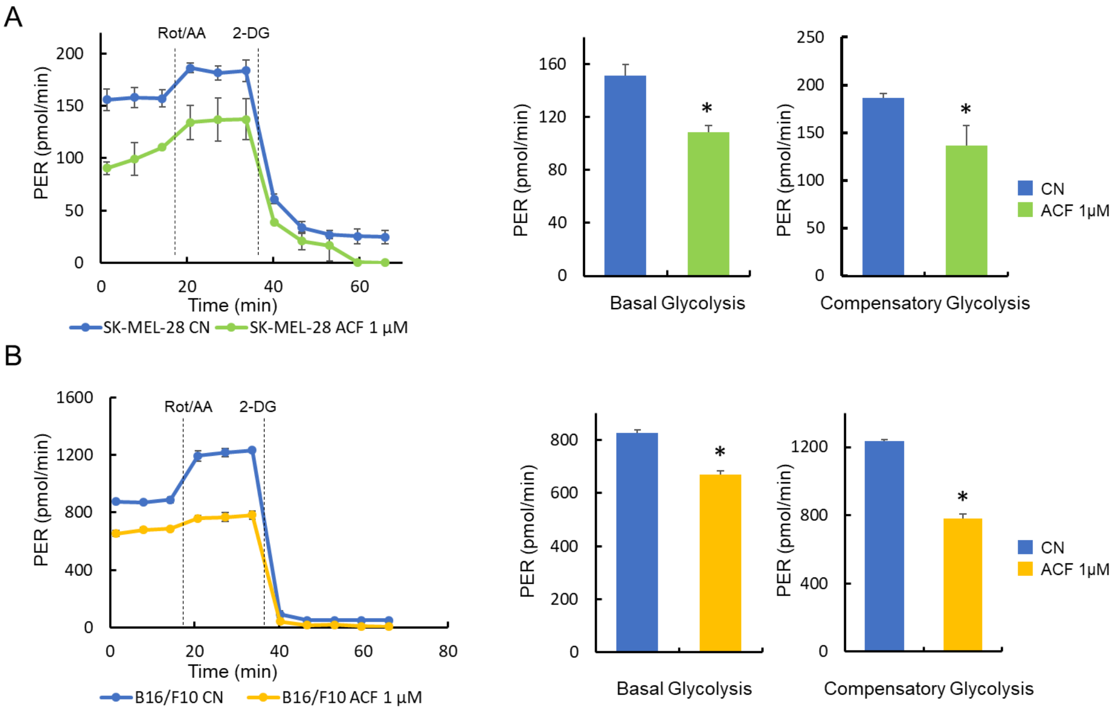

Figure S2. Glycolytic proton efflux rate (glycoPER) comparing untreated and ACF-treated melanoma cells. (A) SK-MEL-28 (B) B16/F10. Assays were performed as indicated in Figure 1F. P values for significant differences (Student's t-test) are summarized by one asterisk ($P < 0.05$) and groups are compared to ACF-untreated samples.

Figure S3. Uncropped Western blots for indicated Figures

Blots from Figure 1

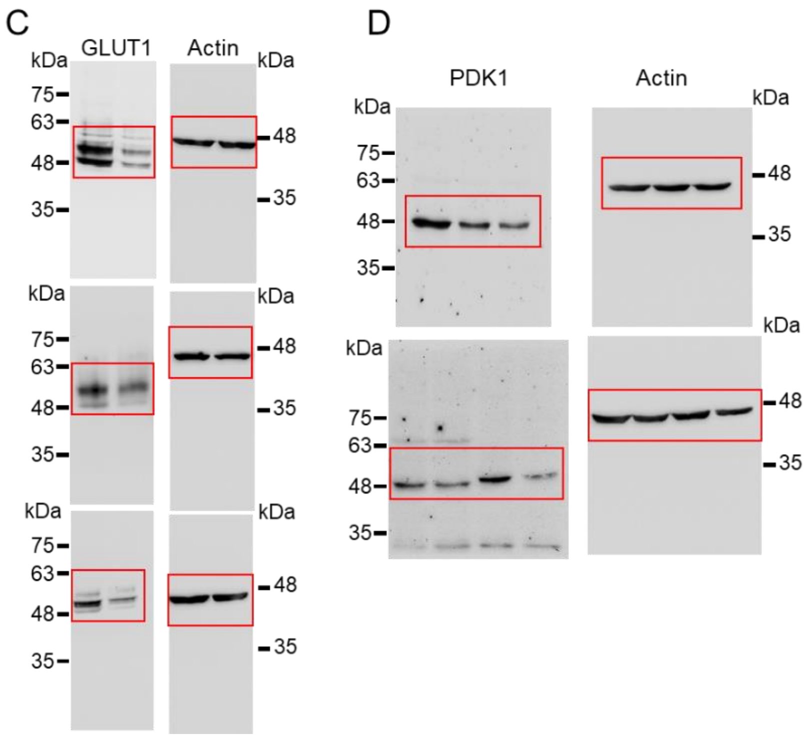

Blots from Figure 2

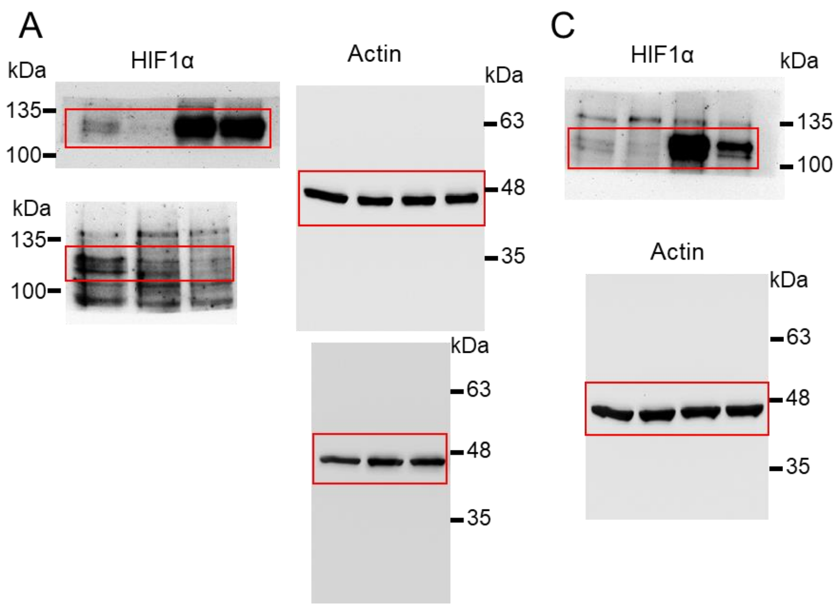

Blots from Figure 3

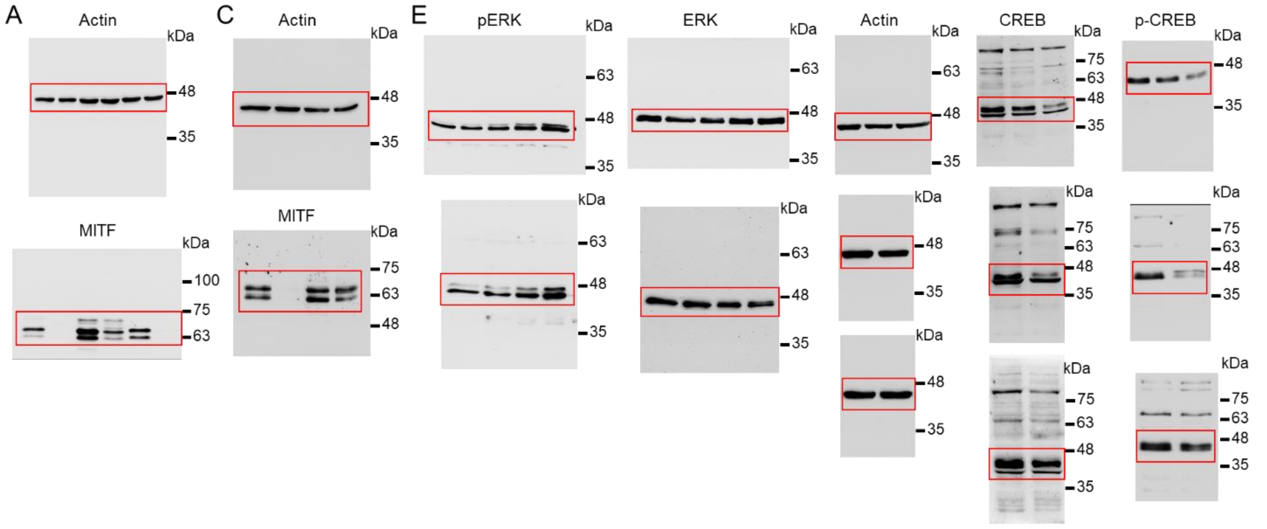

Blots from Figure 4 (A-E)

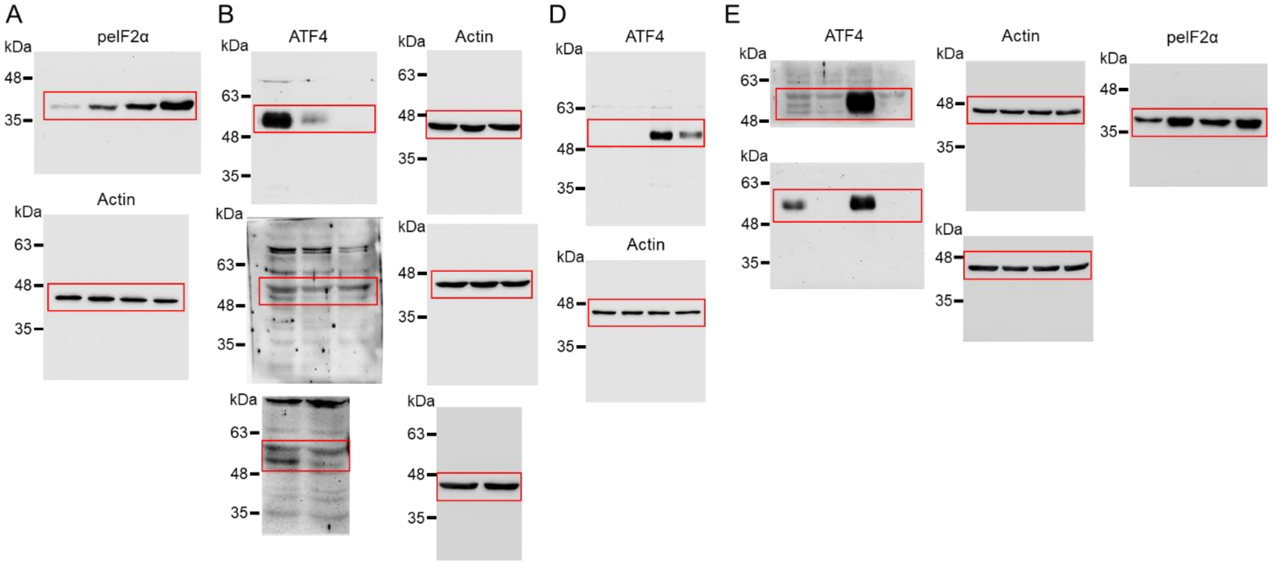

Blots from Figure 4 (F-G)

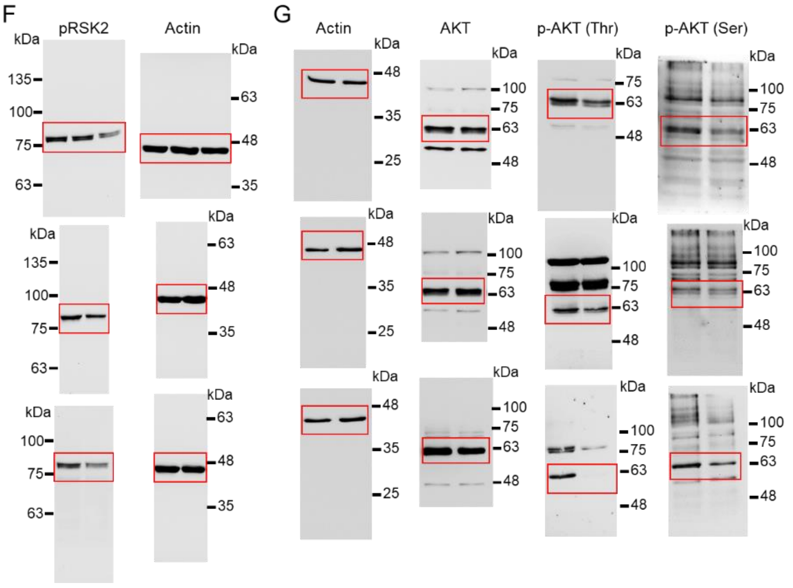

Blots from Figure 5

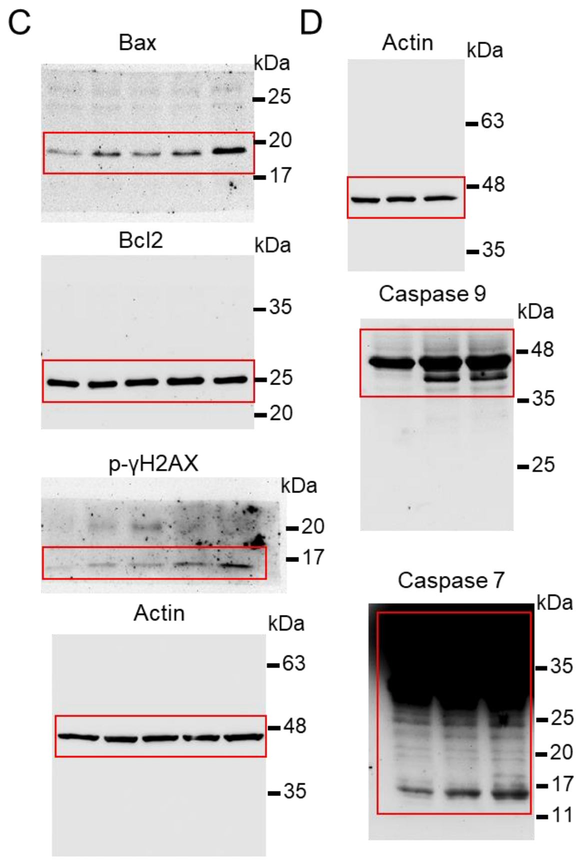

Table S1. Used primary antibodies.

Antibody	Source	Catalogue number
Mouse monoclonal anti-β-Actin (AC-15)	Merck/Sigma	Cat# A5441
Rabbit polyclonal anti-pCREB (Ser133)	Merck/Millipore	Cat# 06-519
Mouse monoclonal anti-pERK1/2	Merck/Sigma	Cat# M9692
Rabbit monoclonal anti-ATF4 (D4B8)	Cell Signaling	Cat# 11815
Mouse monoclonal p-γH2AX (Ser139)	Merck/Millipore	Cat# 05-636
Mouse monoclonal anti-MITF	Merck/Millipore	Cat# MAB3747
Mouse monoclonal anti-BCL2	Merck/Sigma	Cat# B 3170
Rabbit polyclonal anti-BAX	Santa Cruz	Cat# sc-493
Mouse monoclonal anti-HIF1α	Novus	Cat# NB100-105
Rabbit monoclonal anti-peIF2α (Ser51)	Cell Signaling	Cat# 3597
Rabbit polyclonal anti-ERK2	Santa Cruz	Cat# sc-154
Rabbit polyclonal anti-CREB	Merck/Millipore	Cat# 06-863
Mouse monoclonal anti-Caspase-9	Santa Cruz	Cat# sc-17784
Rat monoclonal anti-Caspase-7	Merck/Sigma	Cat# C 1104
Mouse monoclonal anti-GLUT1	Abcam	Cat# ab40084
Rabbit monoclonal anti-p-RSK2	Abcam	Cat# ab75820
Mouse monoclonal anti-p-AKT (Thr308)	Santa Cruz	Cat# sc-271966
Mouse monoclonal anti-p-AKT (Ser473)	Merck/Millipore	Cat# 05-1003
Rabbit monoclonal anti-AKT	Abcam	Cat# ab200195
Mouse monoclonal anti-PDK1	Thermo Fisher	Cat# MA5-15797

Table S2. The following primers for human genes were used.

Genes	Source
MITF RT qPCR primers	Thermo-Fisher
F 5′-3′: GCGCAAAAGAACTTGAAAAC	
R 5′-3′: CGTGGATGGAATAAGGGAAA	
ATF4 RT qPCR primers	Thermo-Fisher
F 5′-3′: TCAAACCTCATGGGTTCTCC	
R 5′-3′: GTGTCATCCAACGTGGTCAG	
PDK1 RT qPCR primers	Thermo-Fisher
F 5′-3′: CGGATCAGAAACCGACACA	
R 5′-3′: ACTGAACATTCTGGCTGGTGA	
HIF1α RT qPCR primers	Thermo-Fisher
F 5′-3′: GAGATGTTAGCTCCCTATATCCCA	
R 5′-3′: TAGGTTCTTGTATTTGAGTCTGCTG	
VEGFA RT qPCR primers	Thermo-Fisher
F 5′-3′: TACTGCCATCCAATCGAGAC	
R 5′-3′: GCATGGTGATGTTGGACT	
β-Actin RT qPCR primers	Thermo-Fisher
F 5′-3′: AGAAAATCTGGCACCACACC	
R 5′-3′: GGGGTGTTGAAGGTCTCAAA	
